# Promoting the Synthesis of Precursor Substances by Overexpressing Hexokinase (Hxk) and Hydroxymethylglutaryl-CoA Synthase (Erg13) to Elevate β-Carotene Production in Engineered *Yarrowia lipolytica*

**DOI:** 10.3389/fmicb.2020.01346

**Published:** 2020-06-19

**Authors:** Shan Qiang, Jing Wang, Xiao Chao Xiong, Yu Ling Qu, Liang Liu, Ching Yuan Hu, Yong Hong Meng

**Affiliations:** ^1^Engineering Research Center of High Value Utilization of Western China Fruit Resources, Ministry of Education, Shaanxi Normal University, Xi’an, China; ^2^National Research & Development Center of Apple Processing Technology, Shaanxi Normal University, Xi’an, China; ^3^College of Food Engineering and Nutritional Science, Shaanxi Normal University, Xi’an, China; ^4^Xi’an Healthful Biotechnology Co., Ltd., Xi’an, China; ^5^Department of Biological Systems Engineering, Washington State University, Pullman, WA, United States; ^6^Department of Human Nutrition, Food and Animal Sciences, College of Tropical Agriculture and Human Resources, University of Hawaii at Manoa, Honolulu, HI, United States

**Keywords:** β-carotene, *Yarrowia lipolytica*, hexokinase, HMG-CoA, glucose utilization

## Abstract

As a valuable carotenoid, β-carotene is commercially used in food, cosmetics, animal feeds, and other industries. Metabolic engineering of microorganisms has been widely explored to improve the production of β-carotene. Compared with the traditional genetic modifications mainly focused on the pathways of mevalonate (MVA) and β-carotene biosynthesis, this study aims to increase the β-carotene production through promoting the synthesis of precursor substances by overexpressing hexokinase and hydroxymethylglutaryl-CoA synthase in an engineered *Yarrowia lipolytica*. In this study, we investigated the effect of the unique hexokinase gene (*Hxk*) overexpression on β-carotene accumulation and glucose consumption. The *Hxk* gene was introduced into a β-carotene producing strain Y.L-1 to generate strain Y.L-2, and this increased the β-carotene content by 98%. Overexpression of the *Hxk* gene led to increasing in hexokinase activity (329% higher), glucose-6-phosphate content (92% higher), and improvement of the transcriptional level of *Hxk* (315% higher) compared to the control Y.L-1 strain. Moreover, *Hxk* overexpression accelerated the utilization rate of glucose. The gene *erg13* encoding hydroxymethylglutaryl-CoA synthase was also overexpressed to increase the precursor supply for β-carotene biosynthesis. Recombinant Y.L-4 harboring two copies of *erg13* produced 8.41 mg/g dry cell weight (DCW) of β-carotene, which was 259% higher than Y.L-1. The β-carotene content of 9.56 mg/g DCW was achieved in strain Y.L-6 by integrating *erg13* into the chromosome and *Hxk* overexpression. The 3-Hydroxy-3-Methylglutaryl-CoA content in the cells was increased by overexpressing two copies of the *erg13* gene. Finally, the titer of β-carotene reached 2.4 g/L using a 50 L bioreactor by the engineered strain, and the fermentation cycle was shortened from 144 to 120 h. Overall, overexpression of *Hxk* and *erg13* could improve β-carotene production and successfully overcoming the bottleneck of precursor generation to support a more efficient pathway for the production of the target product. Our results revealed a novel strategy to engineer the pathway of β-carotene synthesis.

## Introduction

β-carotene is a valuable terpenoid that has broad applications in the food industry as food additives, especially as colorants, as well as in the nutraceutical industry as nutritional supplements (Zhao et al., 2013; Ma et al., 2019). β-carotene is an antioxidant and a precursor for the formation of vitamin A. Compared to chemical synthesis and extraction from plants, microbial biosynthesis of β-carotene is a promising way for sustainable production because of its low production cost and environment-friendly process.

The model organisms, including *Escherichia coli* and *Saccharomyces cerevisiae*, were commonly used host strains for heterologous expression of the β-carotene biosynthesis pathway (Kim et al., 2006; Xie et al., 2013). *Yarrowia lipolytica* has emerged as a new microbial chassis for metabolic engineering as it can use multiple carbon sources for growth and has high carbon flux toward acetyl-CoA (Athanasios et al., 2008; Beopoulos et al., 2009). However, *Y. lipolytica* does not produce β-carotene naturally. Thus, to allow *Y. lipolytica* to produce β-carotene, the genes for biosynthesis of β-carotene need to be introduced into this strain. The genes used to construct of β-carotene biosynthesis pathway include *carB* encoding phytoene dehydrogenase and *carRP* or *carRA* encoding phytoene synthase/lycopene cyclase from natural producers, such as *Mucor circinelloides*, *Xanthophyllomyces dendrorhous*, and *Blakeslea trispora* (Gao et al., 2017; Larroude et al., 2017). The main strategies to promote β-carotene production are to strengthen the mevalonate (MVA) pathway in yeast and the β-carotene biosynthesis pathway by overexpressing the key biosynthesis genes. In the past, β-carotene production has been successfully improved by introducing multiple copies of all the four genes containing truncated hydroxymethylglutaryl-CoA reductase gene (*tHMG*), GGPP synthase gene (*GGS1*), *carRA*, and *carB* in *Y. lipolytica* (Gao et al., 2017).

Typically, glucose metabolism supplies the carbon skeleton of β-carotene and ATP, NAD(P)H using in biosynthesis. The enhancement of glucose consumption by optimizing the media components has increased the β-carotene yield (Larroude et al., 2017). Genetic engineering also can promote glucose utilization capacities. A crucial gene *Hxk* encodes the unique hexokinase that catalyzes the phosphorylation of glucose in the first step of glycolysis. Hexokinase also serves as the initial step in *de novo* biosynthesis of β-carotene (Fickers et al., 2005). Growth of engineered *Y. lipolytica* with deleted *Hxk* was impaired using glucose-based media (Petit and Gancedo, 1999). In *S. cerevisiae*, *Hxk* deletion decreased the maximal glucose consumption rate by 26% and resulted in a decrease in enzyme activity (Miskovic et al., 2017). On the contrary, introducing an additional copy of *Hxk* in *Y. lipolytica* resulted in the improvement of both biomass yield and lipid production (Lazar et al., 2014). Cells in large-scale fermentation always suffer from the low energy level attributed to dissolved oxygen. Therefore, it encourages us to investigate the impact of change in hexokinase activity on glucose utilization rate and β-carotene productivity.

**FIGURE 1:**
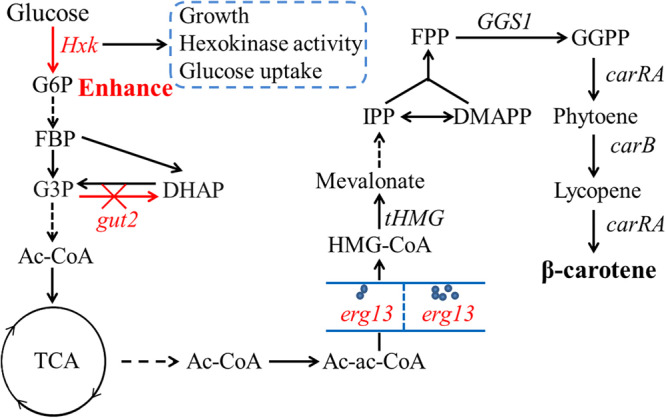
Scheme of the β-carotene synthesis pathway in engineered *Y. lipolytica*. G6P, Glucose 6-phosphate; FDP, Fructose 1,6-bisphosphate; DHAP, Dihydroxyacetone phosphate; G3P, Glyceraldehyde 3-phosphate; PYR, Pyruvate; TCA, Tricarboxylic acid cycle; Ac-CoA, Acetyl coenzyme A; Ac-ac-CoA, Acetyl-acetyl coenzyme A; HMG-CoA, 3-hydroxy-3-methylglutaryl-CoA; IPP, Isopentenyl pyrophosphate; DMAPP, Dimethylallyl pyrophosphate; FPP, Farnesyl pyrophosphate; GGPP, Geranylgeranyl pyrophosphate.

Engineering β-carotene biosynthesis-related genes *tHMG* and *GGS1* is another approach used to promote β-carotene production. The enzymes encoded by these genes can generate the precursor 3-Hydroxy-3-methylglutaryl coenzyme A (HMG-CoA) and farnesyl diphosphate (FPP) in the MVA pathway to biosynthesize β-carotene. HMG-CoA reductase was generally considered as a limiting step in the mevalonate pathway. Thus, additional HMG-CoA reductase gene (*HMGR*) or *tHMG* was overexpressed to elevate carotenoid production (Falk et al., 2014; Gao et al., 2017; Larroude et al., 2017; Schwartz et al., 2017). In the β-carotene biosynthesis pathway (Figure 1), HMG-CoA synthase (HMGS), encoded by *erg13*, transforms the upstream acetyl-acetyl CoA (Ac-ac-CoA) into HMG-CoA, which is the substrate of HMGR. Therefore, in the engineered *Y. lipolytica* overexpressing *tHMG*, *erg13* could be overexpressed to enhance the production of β-carotene. Moreover, the lipid body plays a role in the storage of intracellular hydrophobic compounds, and this property has been used to regulate the production of lipophilic terpenoids (Nambou et al., 2015; Larroude et al., 2017; Zeng et al., 2018). The gene *gut2* encoding glycerol-3-phosphate dehydrogenase has been knockout to improve lycopene storage capacity by increasing the lipid accumulation by preventing the reduction of glycerol-3-phosphate (G3P) (Falk et al., 2014). Therefore, combinational overexpression of the key genes in the β-carotene biosynthesis pathway and engineering of lipid biosynthesis may improve the yield of β-carotene in *Y. lipolytica*.

In this study, we found that *Hxk* (YALI0B22308g) was a useful gene that promotes the glucose utilization and generation of precursors for β-carotene biosynthesis in *Y. lipolytica*. Then we overexpressed the *erg13* (YALI0F30481g) gene with a different copy number of involved in the MVA pathway. Finally, the stable strain was constructed by *gut2* (YALI0B02948g) replaced with the expression cassette of *erg13* in *Y. lipolytica* chromosome and fermentation was also carried out by using the resultant strain. Here the identification of the *Hxk* gene by increasing the glucose consumption provides a new target to engineer metabolically *Y. lipolytica* for β-carotene biosynthesis. The reconstitution of multiple related genes of the β-carotene biosynthesis pathway in *Y. lipolytica* represents the first step for large-scale fermentation to produce β-carotene.

## Materials and Methods

### Strains and Media

All strains and plasmids used in this study were listed in Supplementary Table S1. *Y. lipolytica* strains were grown at 28°C for shake-flask culture on YPD medium containing 10 g/L yeast extract, 20 g/L peptone, and 20 g/L glucose. The yeast transformants were screened on synthetic complete (SC) medium containing 1.7 g/L yeast nitrogen base (YNB) (without amino acids and ammonium sulfate), 5 g/L (NH_4_)_2_SO_4_, 20 g/L glucose, and supplemented with appropriate amino acid dropout mix. SD-leu contained 2 g/L dropout mix synthetic minus leucine, and SD-ura contained 2 g/L dropout mix synthetic minus uracil (US Biological; Marblehead, United States). *Escherichia coli* DH5α was used as gene clone strain and grown at 37°C on LB medium (5 g/L yeast extract, 10 g/L peptone, and 10 g/L glucose) supplemented with 100 μg/mL ampicillin as necessary.

The bioreactor fermentation was performed in a 50 L bioreactor (GRJ-50D, Zhenjiang Green Bioengineering; Jiangsu, China) containing 30 L fermentation medium [25 g/L glucose, 10 g/L yeast extract, 15 g/L peptone, 5 g/L (NH_4_)_2_SO_4_, 2.5 g/L KH_2_PO_4_, 2.5 g/L K_2_HPO_4_, 0.5 g/L MgSO_4_, 6 g/L leucine, 1 g/L biotin]. One single loop of yeast was inoculated in a shake flask containing 200 mL fermentation medium. The flask was cultured for 48 h, and then 700 mL pre-culture was inoculated in the fermenter. Glucose solution (75%) was added to keep the concentration constant (5–10 g/L). One hundred fifty milliliter yeast extract (45 g) and (NH_4_)_2_SO_4_ (30 g) were added every 8 h. Two grams of biotin were added every 20 h. The temperature was maintained at 30°C, and the pH was maintained at 5.8 using NH_3_⋅H_2_O. Oxygen was supplied in the form of filtered air via a sparging rate of 30–50 L/min of air using agitation in the 100–650 rpm range to maintain dissolved oxygen levels above 10–20%.

### Plasmids and Strains Construction

Primers used in this work were listed in Supplementary Table S2. Native genes were obtained through PCR amplification by using *Y. lipolytica* genome DNA as a template. The vector pJN44 (P_TEF_-Txpr2) was used for the expression of genes (Wang et al., 2016). DNA fragments obtained by PCR or enzyme digestion were recovered by using a kit (Axygene; NY, United States). The Seamless Cloning and Assembly Kit (TransGen Biotech; Beijing, China) was used for the construction of plasmids.

Plasmids or DNA fragments were transformed into *Y. lipolytica* at the early stationary phase using Zymogen Frozen EZ Yeast Transformation Kit II (Zymo Research; CA, United States). The detailed method of plasmids and strains construction can be found in Supporting Information.

### Measurement of Hexokinase Activity

Hexokinase (HK) activity was measured as previously described (Acosta et al., 2014). After 96 h of shake-flask culture in the YPD medium, 1 mL of the culture was centrifuged to harvest the cells. The pellet was washed three times with pre-cooled saline and then was subjected to liquid nitrogen grinding to lyse the cell wall with repeated three times. Then the mixture was suspended in 1 mL PBS and centrifuged for at 4°C, 12,000 × g for 5 min to obtain the cell-free extract by discarding the cell debris. HK activity was assayed by measuring the formation of NADPH at 340 nm and 25°C using a spectrophotometer. The formation of NADPH was through coupling the phosphorylation of glucose to the reduction of NADP^+^ by glucose-6-phosphate dehydrogenase (G6PDH). The assay was performed in a 1 mL cuvette containing 1M Tris-HCl, 685 μL pH 7.8, 0.1 M MgCl_2_ 50 μL, 1M ATP 50 μL, 0.08 M D-Glucose 50 μL, 0.08 M NADP^+^ 90 μL, 2 KU/mL G6PDH 50 μL, 1 M DTT 25 μL. The components were mixed for 30 s to detect the enzyme activity. One unit of HK was defined as the amount of enzyme that catalyzed 1 μmole NADP^+^ reduction per minute.

### Glucose, ATP, G6P, and HMG-CoA Determinations

During the cell cultivation, the concentration of glucose was quantified every 24 h using an SBA-40C bioanalyzer (Shandong Academy of Sciences; Shandong, China) according to the instructions.

The intracellular ATP concentration was measured following the instruction of an ATP Assay Kit (Beyotime; Shanghai, China). Briefly, 4 mL-cell cultures in YPD medium were collected every 24 h by centrifuging at 2000 × g for 5 min at 4°C, then thoroughly washing the cell with pre-cooled 0.85% saline. For ATP extraction, 500 μL of ATP detection lysis buffer was added to the cell pellet, vortexed for 15 s, lysed on ice for 5 min, and then centrifuged at 12,000 × g for 5 min at 4°C to obtain the supernatant. The ATP concentration in the supernatant was measured by Synergy H1 Multi-Mode Reader (Gene, Synergy^TM^ H1; Hong Kong, China) (Shi et al., 2016).

G6P in the cells was obtained in the same manner as the isolation of HK. The content of G6P in the supernatant was measured using a G6P Assay Kit with WST-8 (Beyotime; Shanghai, China). The absorbance was measured using a spectrophotometer at 450 nm.

HMG-CoA in the cells was obtained in the same manner as the isolation of HK. The content of HMG-CoA in the supernatant was measured using HMG-CoA Enzyme-Linked Immunosorbent Assay (ELISA) Reagent Kit (Mbbiology; Jiangsu, China). The Kit used a double-antibody sandwich assay to determine the level of HMG-COA in the specimen. The absorbance was measured using ELISA analytical instruments SpectraMax 190 (Thermo Fisher Scientific, CA, United States) at a wavelength of 450 nm. The final value was expressed as ng/g protein. The protein concentration was measured by an enhanced BCA protein assay kit (Beyotime; Nanjing, China).

### Reverse Transcription and Quantitative PCR Analysis

Yeast strains were cultivated for 72 h in the YPD medium. Total RNA was extracted as the methods described previously (Su et al., 2018). RNA samples were reverse transcribed using EasyScript One-Step gDNA Removal and cDNA Synthesis SuperMix Kit (TransGen; Beijing, China) according to the instructions. qPCR analyses were performed using a CFX96 Touch^TM^ Real-Time PCR Detection System (Bio-Rad, CA, United States). The result was normalized to the *actin* gene expression and then analyzed by the 2^–ΔΔCt^ method (Lazar et al., 2011).

### Extraction and Quantification of β-Carotene

All engineered strains were cultured in the YPD medium for the 144 h of shake-flask fermentation. The cells were harvested every 24 h for the measurement of cell growth, dry cell weight (DCW), and β-carotene. One milliliter culture broth was used for the measurement of DCW. β-carotene was extracted as described previously with minor modifications (Gao et al., 2017). In brief, 1 mL cells were harvested by centrifuging at 12,000 × g for 5 min. The obtained cells were re-suspended in 0.5 mL dimethyl sulfoxide, and then incubated for 15 min at 55°C followed by 45°C for 15 min after an equal volume of acetone was added. The samples were then centrifuged at 12,000 × g for 5 min. Supernatants containing β-carotene were filtered through a 0.45 μm filter. The β-carotene analysis was performed by high-performance liquid chromatography (HPLC, Agilent Technologies 1260 Infinity Series System, CA, United States) with the UV signal at 450 nm and a C18 column (4.6 mm × 250 mm). The mobile phase consisted of 50% acetonitrile, 30% methanol, and 20% isopropanol (v/v/v), and the flow rate was 1 mL/min at 30°C. The standard β-carotene was purchased from Sigma-Aldrich (Darmstadt, Germany).

### Statistical Analysis

All data were expressed as Mean ± SD, and each value was the mean of three independent experiments. All data were analyzed by one-way analysis of variance (ANOVA), and significance was determined using the Least Significant Difference (LSD).

## Results

### Overexpression of the *Hxk* Gene Increases β-Carotene Biosynthesis in *Y. lipolytica*

**FIGURE 2:**
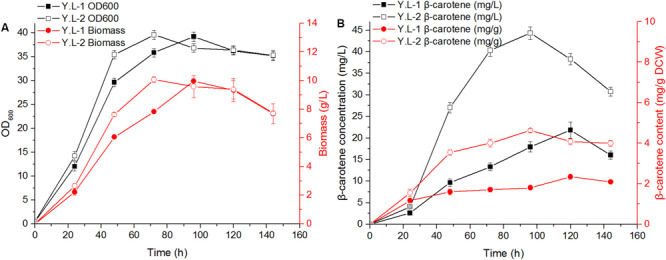
The growth characteristics and β-carotene content of the control strain Y.L-1 and the *Hxk* overexpression strain Y.L-2 in the YPD medium for 144 h shake-flask fermentation. **(A)** The growth characteristics analysis of Y.L-1 and Y.L-2 in the YPD medium for 144 h. **(B)** The β-carotene content of Y.L-1 and Y.L-2 in the YPD medium for 144 h. Error bars represent standard deviations (*n* = 3).

The β-carotene biosynthesis pathway and engineered strategies of *Y. lipolytica* are shown in Figure 1. The basal β-carotene producing strain Y.L-1 was constructed by integrating *tHMG* (YALI0E04807g), *GGS1* (YALI0D17050g), *carRA* (KY971027), and *carB* (KY971026) genes into the *Y. lipolytica* PO1f genome. Among them, the *carRA* and *carB* genes from *B. trispora* were codon-optimized. The genotype of Y.L-1 is listed in Supplementary Table S1. To investigate the effect of the *Hxk* gene on β-carotene production, we introduced an additional copy of *Hxk* under a strong constitutive TEF promoter. The plasmids pJN44-*Hxk* was transformed into Y.L-1 to generate Y.L-2. We compared the growth and β-carotene production by Y.L-1 and Y.L-2 strains. The OD_600_ and biomass of Y.L-1 and Y.L-2 are shown in Figure 2A during 144 h fermentation. Y.L-2 reached the stable phase of growth with an OD_600_ of 39.57 after 72 h of cultivation, whereas Y.L-1 was 96 h. The specific growth rate (μ = 0.0269 h^–1^) of Y.L-2 was different from the specific growth rate (μ = 0.0187 h^–1^) of Y.L-1 in the exponential growth. This result indicated that *Hxk* overexpression caused the cells to grow faster than Y.L-1 and may help to shorten the fermentation cycle. However, *Hxk* overexpression did not lead Y.L-2 to increase biomass production significantly.

β-carotene was extracted and measured to investigate the effect of *Hxk* overexpression on β-carotene production. The β-carotene production of Y.L-1 and Y.L-2 was shown in Figure 2B during 144 h fermentation. Y.L-2 produced 4.63 mg/g DCW of β-carotene, which was 98% higher than that of Y.L-1 (2.34 mg/g DCW).

### Overexpression of *Hxk* Enhances Hexokinase Activity and G6P Content in *Y. lipolytica*

**FIGURE 3:**
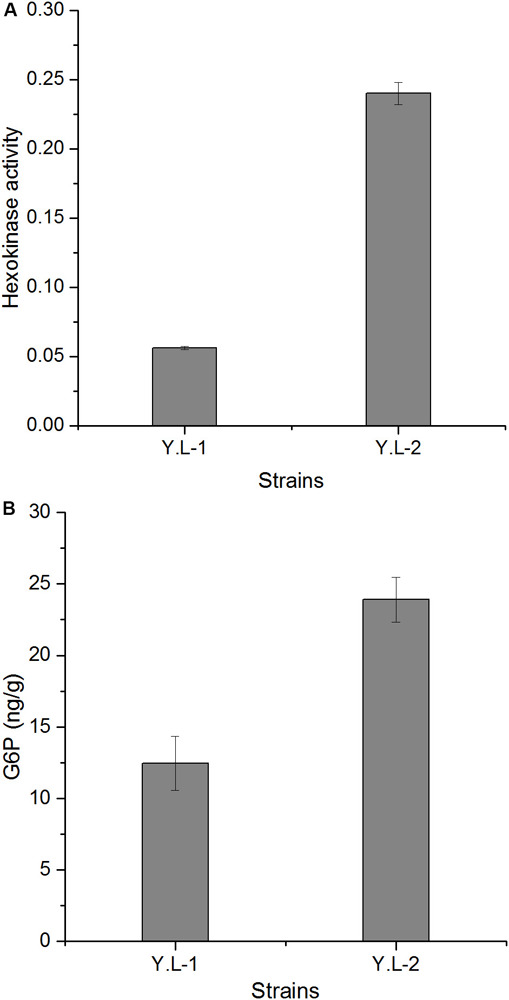
Hexokinase activity and G6P content of the control strain Y.L-1 and the *Hxk* overexpression strain Y.L-2 in YPD medium after 96 h of shake-flask fermentation. **(A)** Hexokinase activity of Y.L-1 and Y.L-2. **(B)** G6P content of Y.L-1 and Y.L-2. Error bars represent standard deviations (*n* = 3).

Hexokinase activity was measured to ensure the overexpression of the *Hxk* gene. As shown in Figure 3A, *Hxk* overexpression increased the HK activity (0.24 mU/OD) in Y.L-2 by 329% than that of Y.L-1 (0.056 mU/OD). This result indicated that *Hxk* was successfully overexpressed. The gene *Hxk* catalyzes the production of glucose-6-phosphate (G6P) with one molecule of glucose. Therefore, we detected the intracellular G6P content to understand the catalytic level of *Hxk*. *Hxk* overexpression enhanced the G6P content (23.91 ng/g) in Y.L-2 by 92% than that of Y.L-1 (12.45 ng/g) (Figure 3B).

### Hexokinase Overexpression Increases the Transcriptional Level of Key Genes in the β-Carotene Synthesis Pathway

**FIGURE 4:**
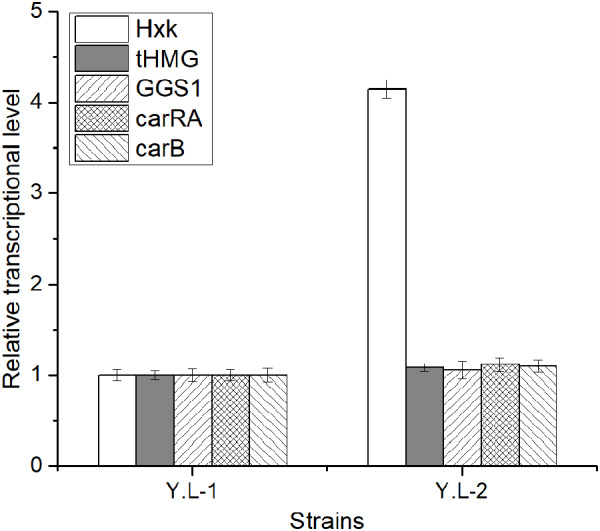
Relative transcriptional level of β-carotene synthesis related genes in the control strain Y.L-1 and the *Hxk* overexpression strain Y.L-2 using real-time PCR in YPD medium after 72 h of shake-flask fermentation. *Actin* was used as the reference gene. Error bars represent standard deviations (*n* = 3).

The transcriptional levels of related genes *Hxk*, *tHMG*, *GGS1*, *carRA*, and *carB* in the β-carotene synthesis pathway were measured separately in Y.L-1 and Y.L-2 strains to investigate the effect of *Hxk* overexpression on their transcription. The results were normalized using the *actin* gene as the internal standard. Compared with Y.L-1, *Hxk* overexpression increased the transcriptional level of *Hxk* by 315% in Y.L-2 (Figure 4), which might be attributed to improving the β-carotene content by 98%. Moreover, *Hxk* overexpression did not significantly increase the transcriptional levels of genes *tHMG*, *GGS1*, *carRA*, and *carB* (*P* > 0.05). Compared to the Y.L-1 strain, the Y.L-2 strain only overexpressed the *Hxk* gene. Therefore, the transcriptional level of *Hxk* in Y.L-2 is up-regulated, while the transcriptional level of genes *tHMG*, *GGS1*, *carRA*, and *carB* is not significantly increased.

### Effect of *Hxk* Overexpression on Glucose Utilization and ATP Content

**FIGURE 5:**
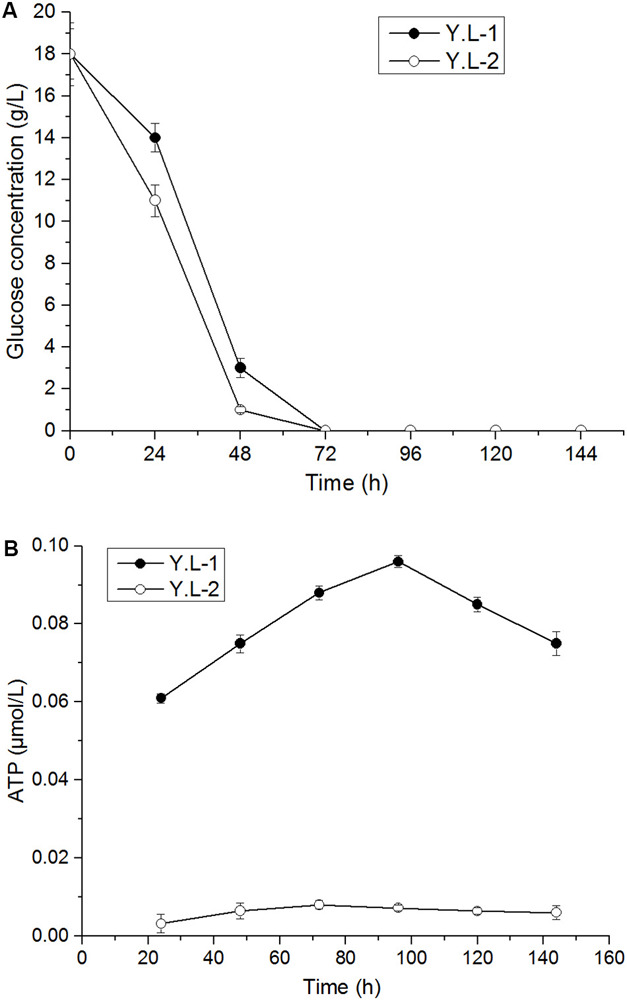
Glucose utilization and ATP content of the control strain Y.L-1 and the *Hxk* overexpression strain Y.L-2 in YPD medium for 144 h shake-flask fermentation. **(A)** The glucose concentration of Y.L-1 and Y.L-2. **(B)** ATP content of Y.L-1 and Y.L-2. Error bars represent standard deviations (*n* = 3).

HK phosphorylates glucose to produce glucose-6-phosphate. Therefore, we measured the utilization of glucose during growth to investigate the effect of HK overexpression on glucose utilization. After 72 h of fermentation (Figure 5A), glucose was completely consumed by Y.L-1 and Y.L-2, but the glucose concentration in the fermentation broth of Y.L-2 was lower than Y.L-1 throughout the fermentation process. In contrast, the *Hxk* overexpression strain Y.L-2 consumed glucose faster (0.35 g/L/h) than the control strain Y.L-1 (0.31 g/L/h) during 48 h of fermentation. This result demonstrated that *Hxk* overexpression increased the catalytic ability of hexokinase and accelerated glucose consumption in *Y. lipolytica* during the fermentation process.

ATP concentration greatly affects cell growth and β-carotene accumulation. For example, Zhao et al. engineered the ATP synthesis module in *E. coli* improving the β-carotene production by 21% (Zhao et al., 2013). In glycolysis, hexokinase catalyzes the phosphorylation of glucose by transferring a phosphate group from ATP to glucose, and this process requires the consumption of ATP. As the most important energy molecule, changes in the ATP level can lead to disorders of the cell. Therefore, it is necessary to determine the intracellular ATP level after the hexokinase overexpression. Within a specific concentration range, the concentration of ATP is proportional to the intensity of fluorescence in the samples. As a result, the *Hxk* overexpression reduced the ATP content in yeast cells throughout the fermentation process (Figure 5B). This result indicates that *Hxk* overexpression requires ATP consumption.

### Further Improvement of β-Carotene Biosynthesis by Overexpressing *erg13* Gene

**FIGURE 6:**
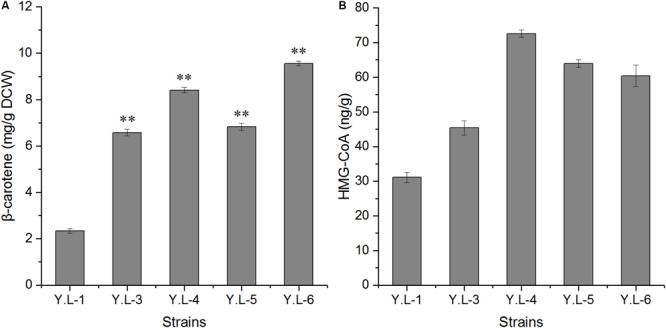
The β-carotene content and HMG-CoA content of the control strain Y.L-1, Y.L-3 overexpressing one copy of *erg13*, Y.L-4 overexpressing two copies of *erg13*, Y.L-5 overexpressing three copies of *erg13*, and Y.L-6 overexpressing *Hxk* and two copies of *erg13* in YPD medium after 96 h of shake-flask fermentation. **(A)** The β-carotene content of Y.L-1, Y.L-3, Y.L-4, Y.L-5, and Y.L-6. **(B)** The HMG-CoA content of Y.L-1, Y.L-3, Y.L-4, Y.L-5, and Y.L-6. Error bars represent standard deviations (*n* = 3). The asterisks indicate a significant difference compared with the control (**p < 0.01).

To increase the accumulation of β-carotene in *Y. lipolytica*, we explored the effect of different copy numbers of *erg13* on β-carotene production. Therefore, plasmids pJN44-*erg13*, pJN44-*erg13-erg13*, and pJN44-*erg13-erg13-erg13* were transformed into Y.L-1 to generate Y.L-3, Y.L-4, and Y.L-5. All the engineered strains are listed in Supplementary Table S1. After 120 h of fermentation, the β-carotene content was compared in Y.L-3, Y.L-4, and Y.L-5 strains. As shown in Figure 6A, Y.L-3, Y.L-4, and Y.L-5 strains produced 6.58 mg/g DCW, 8.41 mg/g DCW, and 6.83 mg/g DCW of β-carotene, respectively (*P* < 0.01). Among the three strains, Y.L-4 overexpressing two copies of *erg13* showed the highest β-carotene content, which was 259% higher than that of Y.L-1. Subsequently, we combined *Hxk* and two copies of *erg13* into Y.L-1. We used the linearized integrative cassette (*erg13-erg13*-*gut2*-up-loxp-down) to insert those two genes into the chromosome of Y.L-1 in the *gut2* site to obtain the genetically stable strain. The detailed method can be found in the supporting information. The obtained strain produced 4.9 mg/g DCW of β-carotene. Then the obtained strain was used for overexpression of pJN44-*Hxk*, resulting in Y.L-6 stain. Y.L-6 produced 9.56 mg/g DCW (Figure 6A) of β-carotene, which was 309% higher than Y.L-1. One of the reasons was that knocking out the *gut2* gene increased lipid content in which β-carotene stored. This result suggests that overexpressing gene *erg13* and glycolysis-related gene *Hxk* and knocking out the *gut2* gene promoted the accumulation of β-carotene in *Y. lipolytica*.

The gene *erg13* catalyzes the production of HMG-CoA with two molecules of Ac-ac-CoA. The increased copy number of *erg13* provides more catalytic substrate HMG-CoA for the step-limiting enzyme HMGR (encoded by *tHMG*). Therefore, we detected the intracellular HMG-CoA content to understand the catalytic level under different copy numbers. As shown in Figure 6B, the HMG-CoA content was 31.1 ng/g protein, 45.41 ng/g protein, 72.58 ng/g protein, and 63.94 ng/g protein in Y.L-1, Y.L-3, Y.L-4, and Y.L-5 strains, respectively. In strains overexpressing different copy numbers of *erg13*, the HMG-CoA content in Y.L-4 overexpressing two copies of the *erg13* gene showed the highest increase, which was 133% higher than that of Y.L-1. The catalytic ability of HMGS in Y.L-4 reached the highest, which might be attributed to improving the β-carotene content by 259%. Notably, the HMG-CoA content was not increased with the three copies number of *erg13* in Y.L-5 (Figure 6B). The HMG-CoA content in Y.L-6 was 60.45 ng/g protein, which was 94% higher than that of Y.L-1. The HMG-CoA content in Y.L-6 was decreased by 17% compared to Y.L-4. The reason may be that the overexpression method of the *erg13* gene is different in Y.L-4 and Y.L-6. Two copies of *erg13* in Y.L-4 were freely overexpressed, while two copies of *erg13* in Y.L-6 was integrated into the Y.L-1 genome and Y.L-6 overexpressed *Hxk*.

### β-Carotene Production by Engineered Strain in a Bioreactor

To further investigate the cell growth and explore β-carotene accumulation characteristics of the engineered strain, the large-scale fermentation experiment was performed using a 50 L bioreactor with the glucose as carbon source and Y.L-1 as control. The engineered strain was obtained by integrating the episomal *Hxk* gene in Y.L-6 into the genome and harboring plasmid pJN44-*tHMG-GGS1-carRA-carB*. The plasmid pJN44-*tHMG-GGS1-carRA-carB* was generated in our laboratory. After the initial 25 g/L glucose was depleted, glucose was fed continuously into the medium to keep its concentration not lower than 5 g/L. We considered the β-carotene content without increasing for 4 h as the endpoint of fermentation. The fermentation experiment was repeated three times, and the results showed that the fermentation was reproducible. We chose one of the fermentation experiments for further analysis.

**FIGURE 7:**
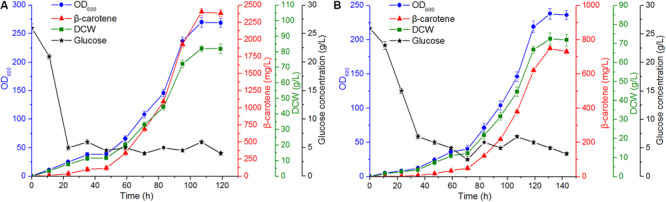
Fermentation characteristics of the engineered strain and the control Y.L-1 strain using a 50 L bioreactor for β-carotene production. **(A)** Fed-batch bioreactor fermentation of the engineered strain for 120 h. **(B)** Fed-batch bioreactor fermentation of the control Y.L-1 strain for 144 h. Error bars represent standard deviations (*n* = 3).

For the engineered strain (Figure 7A), 550 g/L or 4.58 g/L/h of glucose was consumed within the 120 h fermentation. Throughout the fermentation, 5.03 and 5.9 g/L/h of the glucose consumption rate was observed during the exponential growth phase (23–95 h) and stationary phase (95–120 h), respectively. The final biomass was achieved at 82.1 g/L. The maximal β-carotene production in the engineered strain was 2.4 g/L, and the β-carotene content reached 29.23 mg/g DCW. The photograph of bioreactor fermentation was shown in Supplementary Figure S1 by the engineered strain. In the fermentation of Y.L-1 (Figure 7B), 550 g/L or 3.82 g/L/h of glucose was consumed within the 144 h fermentation. Throughout the fermentation, 4.7 and 3.65 g/L/h of the glucose consumption rate was observed during the exponential growth phase (35–119 h) and stationary phase (119–144 h), respectively. The final biomass was achieved at 72.4 g/L. The maximal β-carotene production in the Y.L-1 strain was 0.75 g/L, and the β-carotene content was 10.36 mg/g DCW. These results demonstrate that the engineered strain accelerates glucose utilization and increases the β-carotene production.

## Discussion

At present, *Y. lipolytica* has excellent potential to produce various molecules of interest, such as organic acids (Rywińska and Rymowicz, 2010), fatty acids (Blazeck et al., 2014), and β-carotene (Gao et al., 2017; Larroude et al., 2017), through metabolic engineering. Among them, β-carotene biosynthesis is a complex process and deserves further study in *Y. lipolytica*.

Hexokinase catalyzes the initial intracellular metabolism of hexoses, such as glucose and fructose, in the glycolytic pathway (Fickers et al., 2005). The *Hxk* gene deletion led to a doubling time 15% longer than the wild-type strain on glucose media (Petit and Gancedo, 1999). Overexpression of *Hxk* increased the yield of lipids by 23–55% (Lazar et al., 2014). Moreover, the fermentation time was reduced to approximately 78 h, compared to 120 h (at least) for the control group, due to the improved growth rate and glucose tolerance (Tai and Stephanopoulos, 2013). Until now, engineering studies of *Hxk* have not been performed to increase the β-carotene production. In this study, the strain Y.L-2 overexpressing *Hxk* gene increased the β-carotene content by 98% compared to Y.L-1. We hypothesized that *Hxk* is the main reason for shortening the fermentation cycle from 144 to 120 h in the large-scale fermentation. However, *Hxk* overexpression accelerated cell growth but did not significantly increase cell biomass. We hypothesized that hexokinase catalyzes the phosphorylation of glucose in the glycolytic pathway, which requires ATP consumption. *Hxk* overexpression increased the transcriptional level of *Hxk* by 315%, hexokinase activity by 329%, and the G6P content by 92%. *Hxk* overexpression reduced the ATP content. We hypothesized that the ATP was mainly used for β-carotene synthesis; thus, less ATP is available for cell growth. Therefore, the ATP synthesis related pathway needs further research in the future.

The *erg13* gene catalyzes two molecules of Ac-ac-CoA to form HMG-CoA. HMG-CoA is the substrate for rate-limiting enzyme HMGR. In this study, we overexpressed different copy numbers of *erg13* and found that Y.L-4 with two copies of *erg13* increased the β-carotene content compared to Y.L-1. Moreover, we measured the intracellular HMG-CoA content, and the HMG-CoA content in Y.L-4 showed the highest increase. This result indicated that the catalytic ability of HMGS in Y.L-4 reached the highest. However, the HMG-CoA content did not increase with three copies of *erg13*. The reason most possibly is that three copies of the *erg13* gene were linked together on the pJN44 plasmid, which may have an impact on the function of the gene. Previous studies showed that *gut2* knocked out improved the contents of both lipid and carotenoids (Dulermo and Nicaud, 2011; Falk et al., 2014). The glycolytic pathway can be divided into two parts: the energy consumption part and the energy storage part. The *gut2* gene catalyzes the conversion glyceraldehyde-3-phosphate (G3P) to dihydroxyacetone phosphate (DHAP). Engineering the lipid metabolic pathway was shown to be an effective way to improve the production of another carotenoid, lycopene, with the highest lycopene yield obtained in *Saccharomyces cerevisiae* (Ma et al., 2019). Therefore, two copies of *erg13* were integrated into the chromosome of Y.L-1 in the *gut2* site. Finally, the β-carotene productivity of 2.4 g/L is obtained by the fermentation. This study provides a promising strategy for increasing the synthesis of β-carotene in *Y. lipolytica*, which is beneficial for large-scale production.

## Data Availability Statement

All datasets presented in this study are included in the article/Supplementary Material.

## Author Contributions

SQ, JW, YQ, and YM designed the study. SQ, JW, YQ, and LL conducted the experiments. SQ, JW, XX, YQ, LL, CH, and YM analyzed the data and wrote the manuscript. All authors contributed to the article and approved the submitted version.

## Conflict of Interest

SQ was employed by Xi’an Healthful Biotechnology Co., Ltd.

The remaining authors declare that the research was conducted in the absence of any commercial or financial relationships that could be construed as a potential conflict of interest.

## Supplementary Material

FIGURE S1 Shows the photograph of 50 L bioreactor fermentation by the engineered strain for β-carotene production.

TABLE S1 Shows the plasmid and strains used in this study.

TABLE S2 Shows the primers used in this study.
