# Supplementary material for: Promoting the Synthesis of Precursor Substances by Overexpressing Hexokinase (Hxk) and Hydroxymethylglutaryl-CoA Synthase (Erg13) to Elevate β-Carotene Production in Engineered Yarrowia lipolytica
Source: Front Microbiol. 2020 Jun 19;11:1346. doi: 10.3389/fmicb.2020.01346 (PMC7316989; doi:10.3389/fmicb.2020.01346)
Supplement: FIGURE S1 — Shows the photograph of 50 L bioreactor fermentation by the engineered strain for β-carotene production. [file Data_Sheet_1.docx]

# Promoting the synthesis of precursor substances by overexpressing hexokinase (Hxk) and hydroxymethylglutaryl-CoA synthase (Erg13) to elevate β-carotene production in engineered *Yarrowia lipolytica*

Shan Qiang^1,2^, Jing Wang^1^, Xiao Chao Xiong^3^, Yu Ling Qu^1^, Liang Liu^1^,

Ching Yuan Hu^1^ and Yong Hong Meng^1*^

^1^ Engineering Research Center of High Value Utilization of Western China Fruit Resources, Ministry of Education; National Research & Development Center of Apple Processing Technology; College of Food Engineering and Nutritional Science, Shaanxi Normal University, 620 West Changan Avenue, Changan, Xian 710119, P. R. China

^2^ Xi’an Healthful Biotechnology Co., Ltd., HangTuo Road, Changan, Xian 710100, P. R. China

^3^ Department of Biological Systems Engineering, Washington State University, Pullman, WA, USA

^4^ Department of Human Nutrition, Food and Animal Sciences, College of Tropical Agriculture and Human Resources, University of Hawaii at Manoa, Honolulu 96822, HI, USA

* Corresponding authors: Yong Hong Meng

Tel.: +086 029 85310517, E-mail: [mengyonghong@snnu.edu.cn](mailto:mengyonghong@snnu.edu.cn)

**Materials and Methods**

**Plasmids construction**

Plasmids pJN44 (P_TEF_-Txpr2) containing the leucine selection marker were used for gene episomal expression in *Y. lipolytica* in the study ([Wang et al., 2016](#_ENREF_2)). *Hxk* and *erg13* genes were PCR amplified from *Y. lipolytica* DNA as the template with primers listed in Supplementary Table S2. The PCR products were inserted into pJN44 with a SmaI/Fast Alkaline Phosphatase (FastAP) digest using Seamless Cloning and Assembly Kit (TransGen Biotech; Beijing, China), resulting in pJN44-*Hxk* and pJN44-*erg13*. For the construction of plasmid pJN44-*erg13-erg13*, the fragment of P_TEF_-*erg13* was obtained through PCR amplification of pJN44-*erg13* and was inserted into a pJN44-*erg13* plasmid with SpeI/FastAP digest. The fragment of P_TEF_-*erg13* was inserted into a pJN44-*erg13-erg13* with SpeI/FastAP digest, resulting in pJN44-*erg13-erg13-erg13*.

Plasmids pLoxp-ura-loxp for gene knockout contained the uracil selection marker surrounded by LoxP sites ([Wang et al., 2016](#_ENREF_2)). The integrated plasmid was constructed using the method as described previously (Yan et al., 2020). The 5′ and 3′ flanking regions of *gut2* was amplified from *Y. lipolytica* DNA with the primers listed in Supplementary Table 2, and the fragments were inserted into the upstream and downstream of a uracil marker in pLoxp-ura-loxp respectively, resulting in p*gut2*-up-loxp-ura-down. To construct integrated plasmid pUra-*gut2*-*erg13*-*erg13*, the fragment of *erg13*-*erg13* was obtained from pJN44-*erg13-erg13* with an XbaI/SpeI digest and was inserted into pUra-*gut2*-*erg13*-*erg13* with a SpeI/FastAP digest.

**Strains construction**

This strain Y.L-1 was constructed by integrating *tHMG*, *GGS1*, *carRA* and *carB* genes into the *Y. lipolytica* PO1f genome (ATCC # MYA-2613). The genotype of Y.L-1 is shown in Supplementary Table 1. The strain Y.L-1 was used for episomal expression of pJN44-*Hxk*, pJN44-*erg13*, pJN44-*erg13-erg13*, and pJN44-erg13-erg13-erg13 using a leucine marker. The integrated plasmid pUra-*gut2*-*erg13*-*erg13* was linearized (*erg13*-*erg13-gut2-*up-loxp-down), inserted into Y.L-1 stain, and the transformants were screened using uracil dropout plate. The obtained stain was used for expression of pJN44-*Hxk*, resulting in Y.L-6 stain.

**Supplementary Table S1. Plasmids and stains used in this study.**

| **Plasmids** | **Relative characteristics** | **Source** |
| --- | --- | --- |
| pJN44 | Expression vector, leu^+^, cEN 1-1, TEF, Introl,  xpr2 terminator | ([Wang et al., 2016](#_ENREF_2)) |
| pJN44-*Hxk* | P_TEF_-*Hxk* | This work |
| pJN44-*erg13* | P_TEF_-*erg13* | This work |
| pJN44-*erg13-erg13* | P_TEF_-*erg13*, P_TEF_-*erg13* | This work |
| pJN44-*erg13-erg13-erg13* | P_TEF_-*erg13*, P_TEF_-*erg13*, P_TEF_-*erg13* | This work |
| pLoxp-ura-loxp | Knock out vector, loxp, ura3, ura3-testR,  ura3-F, loxp, AmpR | ([Wang et al., 2016](#_ENREF_2)) |
| p*gut2*-up-loxp-ura-down | *gut2*-up, Loxp, ura3, ura3-testR, ura3-F, loxp, *gut2*-down, AmpR | This work |
| pUra-*gut2*-*erg13*-*erg13* | △*gut2*::*erg13-erg13* in vector p*gut2*-up-loxp-ura-loxp-down, AmpR | This work |
| **Stains** | **Characteristics** | **Source** |
| *E.coli* DH5α | *endA1 hsdR17* [r-m+] *supE44 thi-1 recA1 gyrA* [NalR] *relA relA1* Δ[*lacZYA-argF*] *U169 deoR* [Ø80Δ (*LacZ*) M15] | Novagen |
| PO1f | MatA, leucine-, uracil-, xpr2-322, axp1-2 | ([Madzak et al., 2000](#_ENREF_1)) |
| Y.L-1 | MatA, leucine-, uracil-, xpr2-322, axp1-,  ∆ku70, ∆snf::tHMG-carB-carRA-ggs1 | This Lab |
| Y.L-2 | Y.L-1, pJN44-*Hxk* | This work |
| Y.L-3 | Y.L-1, pJN44*-erg13* | This work |
| Y.L-4 | Y.L-1, pJN44*-erg13-erg13* | This work |
| Y.L-5 | Y.L-1, pJN44-*erg13-erg13-erg13* | This work |
| Y.L-6 | Y.L-1, Δ*gut2*::*erg13-erg13*, pJN44-*Hxk* | This work |

**Supplementary Table S2. Primers used in this study.**

| Primers | | Sequences (5’-3’) |
| --- | --- | --- |
| *Hxk*-For | CAGGTCGACTCTCCCATGGTTCATCTTGGTCCCCGA | |
| *Hxk*-Rev | ACTATCTGTTAACCCCTAAATATCGTACTTGACACCGGGCT | |
| *erg13*-For | CAGGTCGACTCTCCCATGTCGCAACCCCAGAACG | |
| *erg13*-Rev | ACTATCTGTTAACCCCTACTGCTTGATCTCGTACTTTCGTCG | |
| *gut2*-up-For | AGCTAGGGCCCGGGTTGGACAATATACAAATG | |
| *gut2*-up-Rev | ATGCAGCATGCTTGTTTGGGGTGGTGGGT | |
| *gut2*-down-For | ATGCGACTAGTCTGTATAGTAAAAGCGTATAGCC | |
| *gut2*-down-Rev | AGCGACATATGTCGTATAACTTGTAAGTATACAGTAACT | |
| *erg13*-For-1 | CACCACCCCAAACAAGGAATTCGAGCTCGGTACCCG | |
| *erg13*-Rev-1 | GACCGCGATCGAATTTCAGGCGGCCGCGAATTC | |
| *Actin*-F | TCCAGGCCGTCCTCTCCC | |
| *Actin*-R | GGCCAGCCATATCGAGTCGCA | |
| *Hxk*-qPCR-F | CAAGAACGGTCAGGAGCTTG | |
| *Hxk*-qPCR-R | TCGGGCTCAGTGGTGTTAAT | |
| *tHMG*-qPCR-R | CTCAGGACGGTATGACACGA | |
| *tHMG*-qPCR-R | GAGTTGAAGGCCTTTCGCAT | |
| *GGS1*-qPCR-R | ATCAAGGTGGACAAGAGCGA | |
| *GGS1*-qPCR-R | CAACAAGCAGCGACGAGTTA | |
| *carRA*-qPCR-R | GCTCTGCTGGCTATCACCTA | |
| *carRA*-qPCR-R | GTCGTCGCAGAATGTACTCG | |
| *carB*-qPCR-R | GACAAGGACAAGCGAGTGAC | |
| *carB*-qPCR-R | CAGGGTCTTCTTGGTCCAGT | |

**Supplementary Figure S1.** The photograph of 50 L bioreactor fermentation by the engineered strain for β-carotene production.


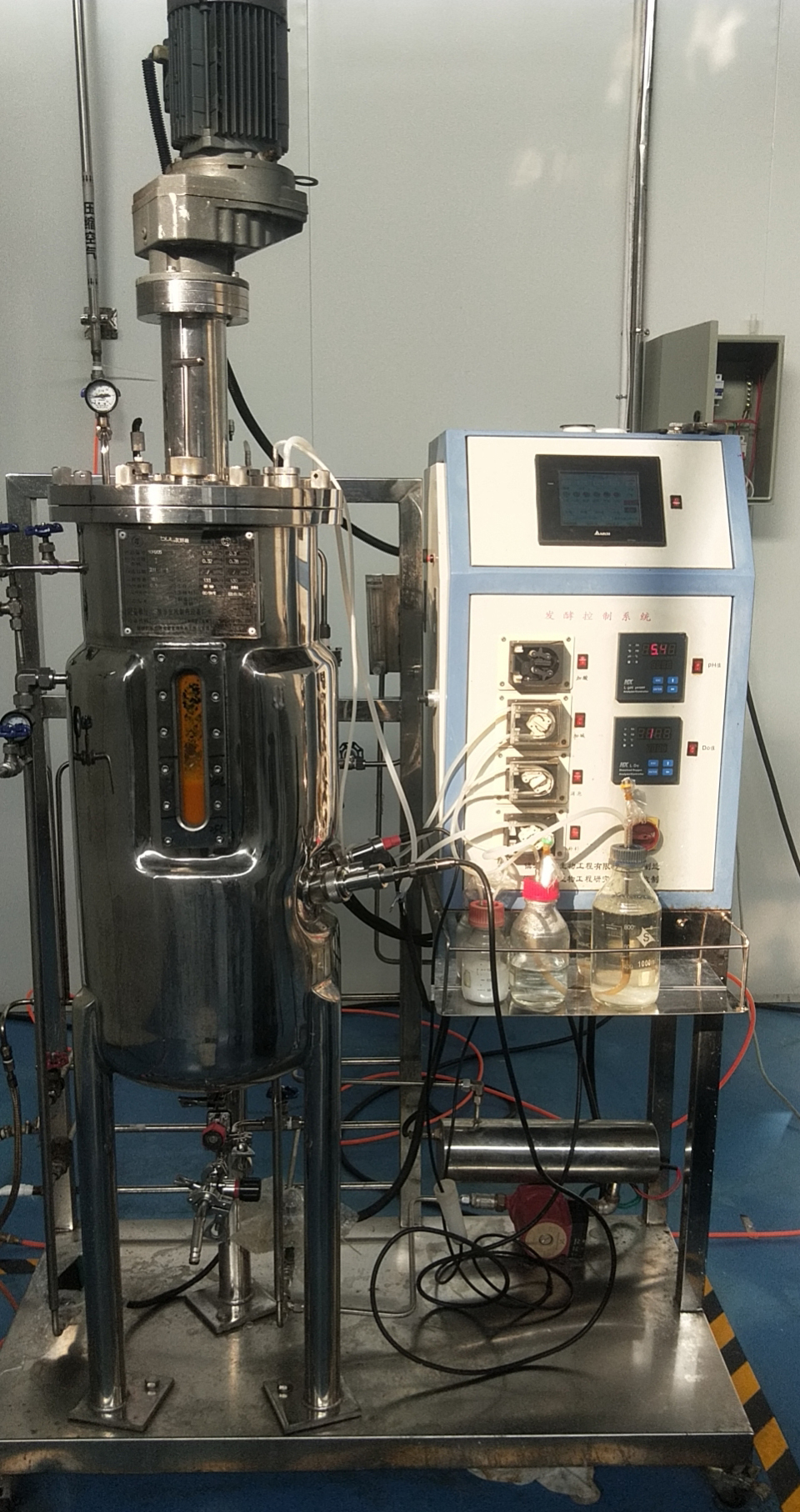

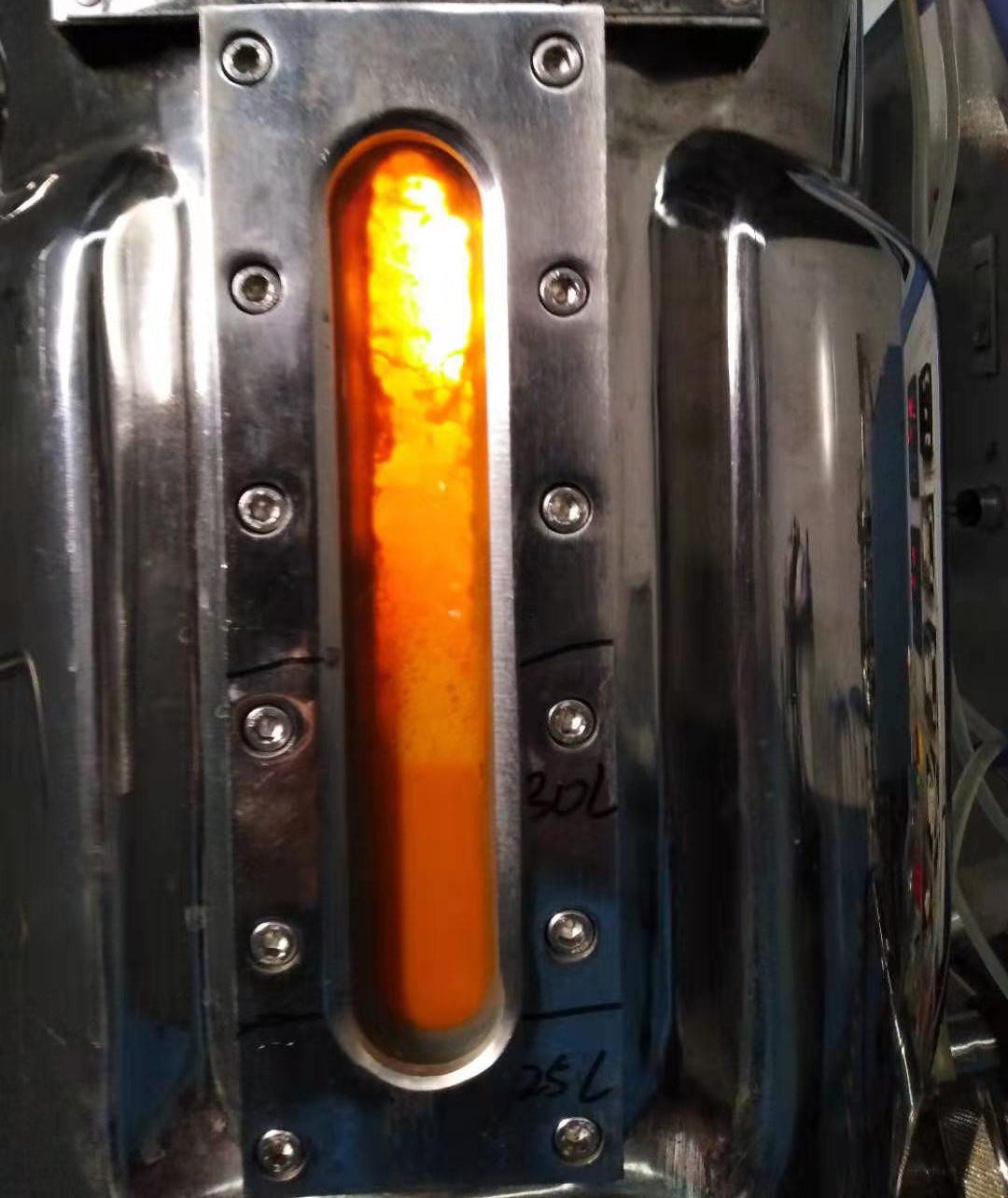


Madzak, C., Tréton, B., and Blanchinroland, S. (2000). Strong hybrid promoters and integrative expression/secretion vectors for quasi-constitutive expression of heterologous proteins in the yeast *Yarrowia lipolytica*. *J. Mol. Microbiol. Biotechnol.* 2, 207-216. doi: [10.1038/sj.jim.2900821](https://doi.org/10.1038/sj.jim.2900821).

Wang, G., Xiong, X., Rishikesh, G., Wang, P., Meng, Y., and Chen, S. (2016). Exploring fatty alcohol-producing capability of *Yarrowia lipolytica*.  *Biotechnol. Biofuels* 9, 1-10. doi: [10.1186/s13068-016-0512-3](https://doi.org/10.1186/s13068-016-0512-3).

Yan, F. X., Dong, G. R., Qiang, S., Niu, Y. J., Hu, C.Y., and Meng, Y. H. (2020). Overexpression of ∆12, ∆15-desaturases for enhanced lipids synthesis in *Yarrowia Lipolytica*. *Front. Microbiol.*2, 289. doi: 10.3389/fmicb.2020.00289.
